# Transcriptome Analyses of Two *Citrus* Cultivars (*Shiranuhi* and *Huangguogan*) in Seedling Etiolation

**DOI:** 10.1038/srep46245

**Published:** 2017-04-07

**Authors:** Bo Xiong, Shuang Ye, Xia Qiu, Ling Liao, Guochao Sun, Jinyu Luo, Lin Dai, Yi Rong, Zhihui Wang

**Affiliations:** 1 College of Horticulture, Sichuan Agricultural University, Chengdu 611130, China; 2 Institute of Pomology and Olericulture, Sichuan Agricultural University, Chengdu 611130, China

## Abstract

*Citrus* species are among the most important fruit crops. However, gene regulation and signaling pathways related to etiolation in this crop remain unknown. Using Illumina sequencing technology, modification of global gene expression in two hybrid citrus cultivars—*Huangguogan* and *Shiranuhi*, respectively—were investigated. More than 834.16 million clean reads and 125.12 Gb of RNA-seq data were obtained, more than 91.37% reads had a quality score of Q30. 124,952 unigenes were finally generated with a mean length of 1,189 bp. 79.15%, 84.35%, 33.62%, 63.12%, 57.67%, 57.99% and 37.06% of these unigenes had been annotated in NR, NT, KO, SwissProt, PFAM, GO and KOG databases, respectively. Further, we identified 604 differentially expressed genes in multicoloured and etiolated seedlings of *Shiranuhi*, including 180 up-regulated genes and 424 down-regulated genes. While in *Huangguogan*, we found 1,035 DEGs, 271 of which were increasing and the others were decreasing. 7 DEGs were commonly up-regulated, and 59 DEGs down-regulated in multicoloured and etiolated seedlings of these two cultivars, suggesting that some genes play fundamental roles in two hybrid citrus seedlings during etiolation. Our study is the first to provide the transcriptome sequence resource for seedlings etiolation of *Shiranuhi* and *Huangguogan*.

*Citrus* is a commercially important genus of the family Rutaceae and widely cultivated fruit crop in the world^1^. Natural and cultivated origin hybrids include commercially important fruit such as the oranges, grapefruit, lemons, some limes, and some tangerines. Both *Shiranuhi (Citrus reticulata* × (*Citrus reticulata* × *Citrus sinenesis*)) and *Huangguogan (Citrus reticulata* × *Citrus sinensis*) are hybrid citrus cultivars, which have been identified as two new cultivated varieties in China. In recent years, the plant area of *Huangguogan* (a new citrus hybrid) and *Shiranuhi* has been expanded rapidly in the southwest of China.

Etiolation, which exists widely in angiosperms, is the phenomenon that plant leaves are yellow when grown in the dark. After germination in the dark, the seedling undergoes etiolated growth referred as skotomorphogenesis, and the leaves display the color of carotenoids. This developmental step is characterized by rapid elongation of the hypocotyl topped by a hook with underdeveloped cotyledons^2^. Changes in plant morphology and growth are the ultimately reflecting of etiolation of plant damage. Etiolation decreased the leaf area, reduced the optical area, and resulting in dwarf plants, weakening growth potential, and even death. Light regulation^3^, ethylene response^4^, riboflavin biosynthesis^5^, endogenous abscisic acid^2^, phospholipid hydroperoxide glutathione peroxidase (PHGPX)^6^, and proteome analysis^7^ have been employed to examine the growth and development of etiolation.

There are far more studies about de-etiolation. The transition from skoto- to photomorphogenesis, called de-etiolation, represents the switch from heterotrophy to autotrophy^8^. As soon as the seedling perceives the light, photomorphogenesis starts. De-etiolation, on the other hand, is a series of morphology, physiological and biochemical changes a plant shoot undergoes in response to sunlight. The changes include those in seedling and physiology, triggered by the light-regulated expression of numerous genes^3^. This process is also known informally as greening of leaves due to chlorophyll formation and chloroplast development. This phenomenon has been studied, including the signaling regulation of expression of nuclear and plastidic genes coding for chloroplast proteins^9^,^10^. On the other hand, only scarce information is available on effects in mitochondria and mitochondrial activity during greening.

Light regulates a wide range of plant processes including seed germination, organ, cell and organelle differentiation, flowering^8^,^11^,^12^,^13^, and metabolism^14^. It is one of the most influential environmental stimuli, which regulates virtually all aspects of growth and developmental processes^15^,^16^. It has been reported that more than 1000 genes are modulated at the transcript level during the greening of *Arabidopsis* and *Rice* etiolated seedlings^17^. There have been so many studies on greening, mainly focused on gene expression, particularly of photosynthetic genes, and on modulation of the corresponding protein levels^18^. In addition, a proteomic analysis that focused on light-induced development of chloroplasts from etioplasts was also performed on rice seedlings during greening^19^. The etioplast to chloroplast transition, another research approach, has focused on the characterization of the processes in the etioplast stroma and some aspects of the assembly of a thylakoid membrane system^19^,^20^,^21^,^22^. All together, these studies have clarified the differences in modulation of distinct metabolic pathways during de-etiolation and provided a broad panel of proteins and some genes, which are potentially associated with de-etiolation or greening.

However, all these descriptions are all at the metabolism regulation, protein translation and resources for genetic improvement.

Transcriptome analysis (RNA-seq) provides a rapid and cost-effective approach to obtain massive protein-coding genes^23^, which can be used for understanding ecological, comparative and evolutionary genomics questions for non-model organisms^24^. In the present study, we illustrate the possible mechanism of etiolation. The RNA-Seq platform was used to analyze the expression profiles of etiolation related genes in three stages of leaves tissue. The identified candidate genes could help to elucidate the molecular basis of etiolation.

## Results

### Transcriptome assembly and annotation

In this experiment, we constructed six cDNA libraries, including R_Y, R_M and R_G, which represent etiolated seedlings, multicoloured seedlings and green seedlings from *Shiranuhi*, respectively, and likewise, Y_Y, Y_M and Y_G, except that they are from *Huangguogan*. After removing sequencing adaptors and low quality data, we obtained 834.16 million clean reads and 125.12 Gb of RNA-seq data, more than 91.37% reads had a quality score of Q30 (sequencing error rate, 0.1%). Statistics of sequencing data is listed in Table 1. All the raw data was deposited into NCBI Gene Expression Omnibus (GEO) with accession number GSE90935.

**Figure 1:**
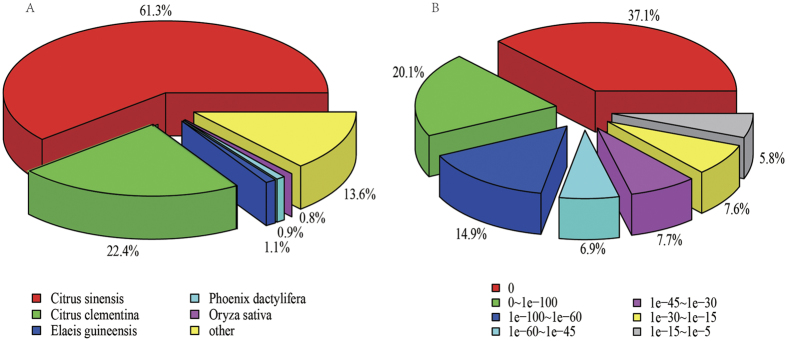
Species classification (**A**) and e-value distribution (**B**) of the unigenes of *Shiranuhi* and *Huangguogan* annotated to NCBI NR database.

Transcriptome *de novo* assembly was performed using Trinity, a short reads assembling program^25^. All together, 205,219 transcripts and 124,952 unigenes were generated. The total length of transcript was 244,060,930 bp with a mean length of 1,189 bp and an N50 of 2,463 bp. The total length of unigene was 221,401,747 bp with a mean length of 1,772 bp and an N50 of 2,690 bp. Detail information is shown in Supplementary Figures S2 and S3. The top species classification hits for *Shiranuhi* and *Huangguogan* in the NR database are *Citrus sinensis* and *Citrus clementina* (Fig. 1A), which all belong to Rutaceae. The e-values are very significant, which mostly close to zero (37.1%) and 0~1e–100 (20.1%) (Fig. 1B), suggesting that most unigenes of *Shiranuhi* and *Huangguogan* have very similar homologs in above two citrus.

After assembly, the 124,952 all-unigenes were subjected to public databases including NR, NT, KOG, Swiss-prot, KEGG and GO using BLAST (E-value ≤ 10^−5^). Eventually, a total of 111,163 (88.96%) unigenes were annotated in at least one database (Supplementary Table S2). Venn diagram of the unigenes was presented in Supplementary Figure S4.

### Gene expression level evaluation

FPKM (fragments per kilobase of exon per million fragments mapped) was used to quantify the expression level of unigenes. The expression level detected by RNA-seq is highly sensitive. Overall distribution of gene expression level of six libraries is shown in Supplementary Figure S5, suggesting that the alteration of gene expression is more visible in *Huangguogan* compared to *Shiranuhi*.

Hierarchical cluster analysis was carried out with 66 significantly differential expressed genes (DEGs) in etiolated and multicoloured seedlings of *Shiranuhi* and *Huangguogan*. Genes with same or similar expression profile were clustered, so as to present differential expressing patterns of gene sets under various experimental conditions. Cluster results of DEGs in six libraries are shown in Supplementary Figure S6.

### Identification of differential expressed genes (DEGs) of etiolation

**Figure 2:**
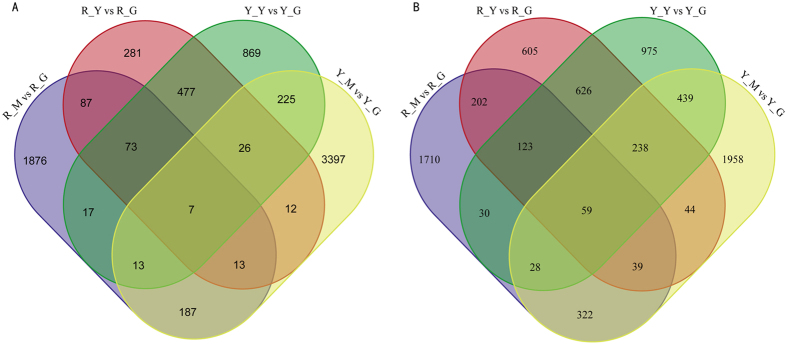
Veen diagram show the number of DEGs between etiolated, multicoloured seedlings of *Shiranuhi* and *Huangguogan*. (**A**) The Venn diagram displays the distribution of up-regulated genes in R_Y vs R_G, R_M vs R_G, Y_Y vs Y_G, and Y_M vs Y_G. (**B**) The Venn diagram displays the distribution of down-regulated genes in R_Y vs R_G, R_M vs R_G, Y_Y vs Y_G, and Y_M vs Y_G.

In the process of DEGs screening, we used *P*-value < 0.005^26^ and log_2_FC (fold change) > 1 as the threshold to determine the significance of gene expression difference. FC is the ratio of FPKM between etiolated and green seedlings. DEG profile analysis was used to analyze gene expression in the two stages of *Shiranuhi* and *Huangguogan* leaf etiolation. The changes in gene expression between etiolated, multicoloured and green seedlings was analyzed in a Venn diagram (Fig. 2), which illustrated the intersections between the expressed genes detected in the two stages of *Shiranuhi* and *Huangguogan* leaf etiolation.

14,958 genes expressed in at least one of the samples. 2912 DEGs between R_Y and R_G, including 976 (33.52%) up-regulated genes and 1936 (66.48%) down-regulated genes. In Y_Y and Y_G, we found 4225 DEGs, among which 1707 (40.41%) were induced and 2518 (59.59%) were suppressed. A total of 4786 DEGs were detected between R_M and R_G, with 2273 (47.49%) up-regulated and 2513 (52.51%) down-regulated. In Y_M and Y_G, we found 7007 DEGs, among which 3880 (55.37%) were induced and 3127 (44.63%) were suppressed. In total, 7 significantly differentially up-expressed genes were found in R_Y, R_M, Y_Y and Y_M (Fig. 2A), and 59 genes were differentially down-expressed (Fig. 2B). 1629 DEGs were found in both *Huangguoga*n and *Shiranuhi*, reflecting the common etiolation in both varieties. When these two cultivars were exposed to etiolation, the number of down-regulated genes (1046 DEGs) was higher than that of up-regulated genes (583 DEGs), and *Huangguogan* had more DEGs than *Shiranuhi* (Fig. 2), indicating more complex etiolation response pathways in *Huangguogan*. This indicated that while many genes were involved in the overall process of etiolation, far fewer genes were functionally unique to the *Citrus* seedling etiolation.

In general, the change of gene expression in *Shiranuhi* is not as noticeable as *Huangguogan*. In R_Y library, log_2_FC of DEGs ranged from −10.68 to 12.18, while in Y_Y library, this parameter fluctuated between −13.04 and 10.52. In R_Y and Y_Y, 25 and 11 DEGs changed at least 500 fold change, respectively. In R_Y, Cluster-2274.31930 was the highest up-regulated gene (log_2_FC = 12.18). It was annotated as unknown protein in KOG. Whereas Cluster-2274.52311 showed the greatest decrease in expression (log_2_FC = −10.68), which encodes multicopper oxidases. In Y_Y, Cluster-2274.39936 exhibited the highest expression level (log_2_FC = 10.52), which encodes transketolase. While Cluster-2274.65696 expression displayed the most dramatic repression (log_2_FC = −13.04) and was also annotated as unknown protein. Despite the function of the highest up and down-regulated gene in each library is unclear, further analysis for functional identification of these genes is needed.

Apart from those genes that showed the greatest changes in expression, some genes with the highest expression level (FPKM value) deserve attention, because these genes may also play important roles in etiolation. Genes with the top ten FPKM in R_Y and Y_Y are listed in Table 2. Six genes were common to both libraries, four of which (Cluster-2274.39471, Cluster-2274.59829, Cluster-2274.79091, Cluster-2274.52612) were associated with protein. They encode asparagine synthase, OmpW family protein, Glycosyl hydrolases family protein and Myo-inositol oxygenase, respectively. One specific gene, Cluster-2274.29764, were related to regulation of transcription, which encode related to No apical meristem (NAM) protein. Another gene, Cluster-2274.64715, had no available description. The expression levels of these six genes were higher in R_Y than Y_Y.

Besides, R_Y and Y_Y owned four unique genes, respectively. In R_Y, three of four specific genes (Cluster-2274.31940, Cluster-2274.31935, Cluster-2274.78324) were related to protein, which encode related to zinc finger (CICLE-type), zinc finger (CICLE-type), and Snf7 family protein, respectively. The other gene, Cluster-2274.47716, had unknown function. In Y_Y, three genes (Cluster-2274.56949, Cluster-2274.48669, Cluster-2274.48671) encode Prolyl oligopeptidase family, hAT family, and BED zinc finger, respectively. Another gene, Cluster-2274.55102, was related to development, which encodes Lea5 (late embryogenesis abundant protein), also known as late embryogenesis abundant protein Lea5-like (*LEA* 5). It has a role on stress tolerance and its mRNA levels are elevated in response to salt, heat and drought stress^27^.

### Functional classification of DEGs

**Figure 3:**
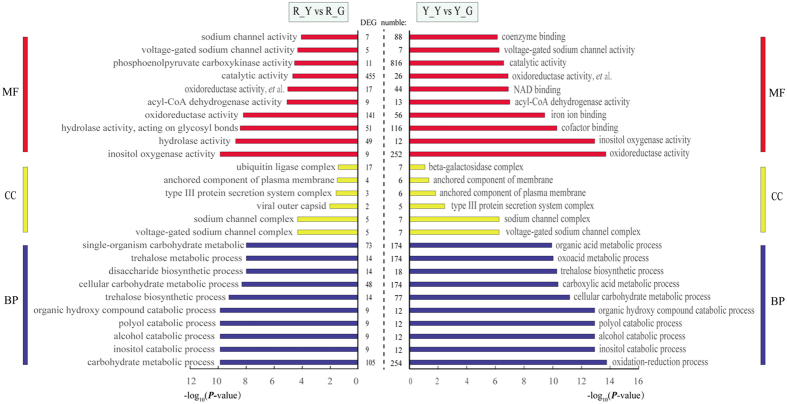
Functional classification of up-regulated DEGs in R_Y and Y_Y. Gene ontology biological process (GO-BP), gene ontology cellular component (GO-CC), and gene ontology molecular function (GO-MF) categories enriched in the up-regulated DEGs. Oxidoreductase activity, *et al*. is the short for oxidoreductase activity, acting on single donors with incorporation of molecular oxygen.

To further highlight the distinct biological function, Gene ontology biological process (GO-BP), gene ontology cellular component (GO-CC), and gene ontology molecular function (GO-MF) categories enriched in the up-regulated DEGs of R_Y and Y_Y libraries. Functional classification of up-regulated DEGs in R_Y and Y_Y are displayed in Fig. 3. The results showed that in both libraries, genes involved in gene ontology biological process (GO-BP), such as inositol catabolic process (GO: 0019310), alcohol catabolic process (GO: 0046164), polyol catabolic process (GO: 0046174), organic hydroxy compound catabolic process (GO: 1901616), cellular carbohydrate metabolic process (GO: 0044262), and trehalose biosynthetic process (GO: 0005992). Genes involved in gene ontology cellular component (GO-CC), such as voltage-gated sodium channel complex (GO: 0001518), sodium channel complex (GO: 0034706), type III protein secretion system complex (GO: 0030257), and anchored component of plasma membrane (GO: 0046658). Genes involved in gene ontology molecular function (GO-MF), such as oxidoreductase activity (GO: 0016491), oxidoreductase activity, acting on single donors with incorporation of molecular oxygen (GO: 0016701), inositol oxygenase activity (GO: 0050113), acyl-CoA dehydrogenase activity (GO: 0003995), catalytic activity (GO: 0003824), and voltage-gated sodium channel activity (GO: 0005248). Therefore, our analysis is focused on these aspects. In these groups, the number of up-regulated genes in Y_Y was higher than R_Y. In R_Y and Y_Y, 1105 (37.95%) and 1329 (31.46%) DEGs were clustered in “not assigned” of gene ontology (GO), respectively. Some of these gene may be novel genes involving in etiolation response that have never been reported.

**Figure 4:**
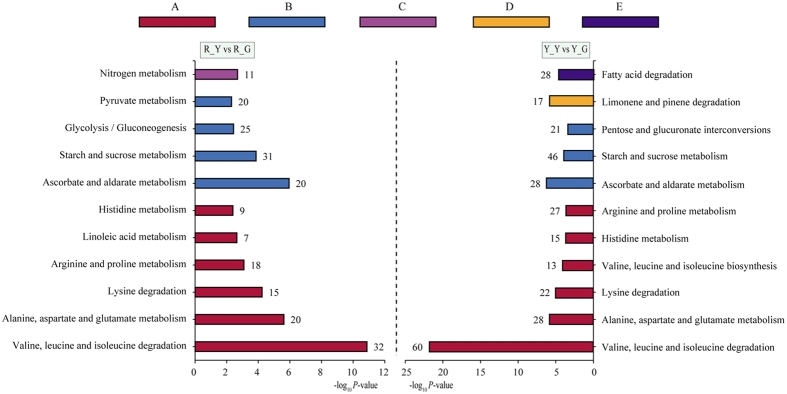
Distribution of KEGG classification of up-regulated DEGs in *Shiranuhi* and *Huangguoga*n. The *P*-value was calculated using the Benjamini correction method and Fisher’s exact test (*P*-value < 0.01). (**A**) Amino acid metabolism. (**B**) Carbohydrate metabolism. (**C**) Energy metabolism. (**D**) Metabolism of terpenoids and polyketides. (**E**) Lipid metabolism.

The distribution of top 11 KEGG pathway of up-regulated DEGs showed that in all annotated amino acid metabolism (101 and 165 DEGs in etiolated *Shiranuhi* and *Huangguoga*n seedlings, respectively) and carbohydrate metabolism (96 and 95 DEGs in etiolated *Shiranuhi* and *Huangguoga*n seedlings, respectively) have the most hits (Fig. 4). These pathways were mainly over-represented in amino acid metabolism in both R_Y and Y_Y. There were 7 common pathways in both R_Y and Y_Y library, it enriched in amino acid metabolism (KO 00280, KO 00250, KO 00310, KO 00330, KO 00340), carbohydrate metabolism (KO 00053, KO 00500). 32 and 60 up-regulated DEGs were mainly over-represented in valine, leucine and isoleucine degradation (KO 00280) of metabolism in R_Y and Y_Y, respectively (Supplementary Tables S3 and S4).

### qRT-PCR validation

**Figure 5:**
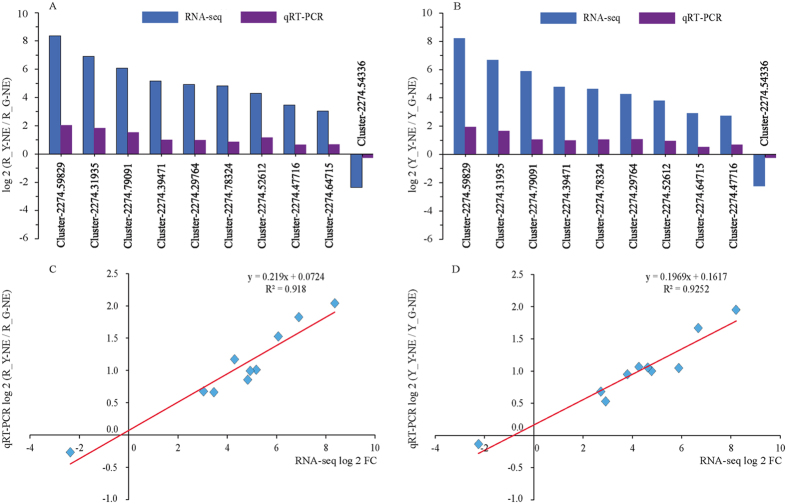
Expression pattern of 10 selected genes as obtained by RNA-seq and qRT-PCR. (**A**) qRT-PCR validation for the 9 randomly selected DEGs of *Shiranuhi*. (**B**) qRT-PCR validation for the 9 up- and 1 down-regulated DEGs of *Huangguogan*. R_Y-NE, R_G-NE, Y_Y-NE and Y_G-NE represent normalized expression levels for the DEGs in the *Shiranuhi* and *Huangguogan* libraries, respectively. FC is the ratio of FPKM between etiolated and green seedlings. (**C**,**D**) Scatter plot of 10 selected genes based on fold change measured by RNA-seq and by qRT-PCR analysis of *Shiranuhi and Huangguogan*, respectively. A linear trend line is shown. Pearson’s correlation was used to determine the relationship between the qRT-PCR and RNA-seq results for DEGs expression levels.

To verify the reliability and accuracy of our transcriptome data, we selected 9 up-regulated and 1 down- regulated unigenes from common DEGs in R_Y and Y_Y libraries and evaluated their expression profiles using quantitative real-time PCR. *Actin*, which is one the most widely used reference genes, was selected for internal controls. The expression patterns of selected genes were determined and further compared with those of in RNA-seq assay. Nearly all of these genes displayed similar expression trend in both techniques (Fig. 5A,B). Moreover, the correlation between qRT-PCR and RNA-seq was measured by scatter plotting log_2_ (R_Y-NE/R_G-NE) and log_2_ FC (Fig. 5C,D), which showed a positive correlation coefficient (Pearson coefficient *R*^2^ = 0.918 and 0.9252, respectively).

## Discussion

### Up-regulated genes and down-regulated genes

When exposed to etiolation, the number of down-regulated genes clearly exceeded that of up-regulated genes in both R_Y, R_M and Y_Y libraries, but it was more up-regulated DEGs in Y_M. On that account, it appears that etiolation is the key point to determine whether up- and down-regulated DEGs are more or less. Taken together, the relative ratio between up-regulated genes and down-regulated genes in response to etiolation may vary with different citrus cultivars. There is no GO: 0009704 in all libraries, in which all genes and gene products annotated to de-etiolation, indicating there is no leaf de-etiolation in. In R_Y and Y_Y, more than 31% DEGs were clustered in “not assigned” of gene ontology (GO), respectively. Some of these gene may be novel genes involving in etiolation response that have never been reported.

During etioplast to chloroplast conversion, there is an increase in proteins related to photosynthesis, Calvin cycle, and proteins involved in translational regulation of gene expression, whereas enzymes involved in amino acid and fatty acid metabolism were decreased in relative abundance^19^. Photosynthesis-related proteins are coregulated with proteins involved in fatty acid metabolism and translational gene expression upon illumination of etiolated rice seedlings^28^. Intriguingly, there was a consistent increase in the levels of isoleucine, glycine, phenylalanine, arginine, and lysine in *Arabidopsis*, i.e. growing the plants in photosynthetic conditions and then depriving them of light^29^,^30^. Plastids perform essential biosynthetic and metabolic functions in plants, including photosynthetic carbon fixation and synthesis of amino acids, fatty acids, starch, and a vast array of secondary metabolites^31^. But there were two down-regulated genes (Cluster-2274.52450, Cluster-2274.62235) in R_Y and Y_Y, which were enriched in chlorophyllide a oxygenase [overall] activity (GO: 0010277), resulted in blocking the synthesis of chlorophyll. At the same time, our data confirm the pathways were mainly over-represented in amino acid metabolism in both R_Y and Y_Y, and the up-regulated DEGs were also involved in starch and sucrose metabolism and fatty acid metabolism (Fig. 4).

In non-photosynthetic tissues NADPH, produced via the oxidative pentose phosphate pathway, is the likely electron donor for ferredoxin reduction^32^,^33^. This study found that two up-regulated genes (Cluster-2274.61951, Cluster-2274.61959) were enriched in pentose phosphate pathway (KO 00030) in both R_Y and Y_Y libraries, but it was not in R_M and Y_M. Our data provide little evidence that pentose phosphate pathway is promoted in response to photosynthesis for leaf etiolation.

### Possible mechanism of etiolation

Based on our transcriptome data, we speculate that there may exist four reasons to explain the etiolation of citrus seedlings. Firstly, some previously reported genes responsible for etiolation were differentially expressed in R_Y, R_M, Y_Y and Y_M. An example is Cluster-2274.55102, was related to development, which encodes Lea5 (late embryogenesis abundant protein), also known as late embryogenesis abundant protein Lea5-like (*LEA 5*). It has a role on stress tolerance and its mRNA levels are elevated in response to salt, heat and drought stress^27^. This gene was up-regulated by 3.66, 4.39 fold in R_Y and Y_Y, respectively, which agrees with its positive regulator role.

In the second place, *Huangguogan* featured higher expression level of some commonly changed genes. For instance, 7 genes (Cluster-2274.11818, Cluster-2274.22872, Cluster-2274.78062, Cluster-2274.35672, Cluster-2274.58920, Cluster-2274.31722, Cluster-2274.48364) were all up-regulated in R_Y, R_M, Y_Y and Y_M libraries, but in Y_Y its expression level was higher than R_Y. The number of DEGs was larger in R_M and Y_M compared to R_Y and Y_Y, respectively (Fig. 2). When blasting in *Citrus Sinensis* Annotation Project (http://citrus.hzau.edu.cn/cgi-bin/orange/blast), found that three of these genes (Cluster-2274.58920, Cluster-2274.31722, and Cluster-2274.48364) were associated with chloroplastic or cytochrome, can’t be enriched to KEGG. For example, Cluster-2274.31722 was associated with detoxifying and antioxidant, it encode ferritin in *R. communis*^34^ and sweet orange^35^ for GenBank blast annotation. All together, there may be a pathway, having not been reported, to response for etiolation. Previous studies have found that there are eight different annexins (*AnnAt1*-*8*) in *Arabidopsis*, some of which are likely to have unique individual functions^36^, the whole annexin gene family contributes importantly to the diverse cellular functions needed for seedling growth. Additionally, plant annexins have been found in cytoplasm, vacuole and nucleus^37^. Plant annexins, which are encoded by the *Ann* genes, have also been implicated in imparting tolerance to various abiotic stresses^38^,^39^. *AnnAt1* was found to be de-etiolated responsive^37^. In present study, more than eleven DEGs were enriched in amino acid transmembrane transport, 8 common DEGs, it can be indicted that some of these DEGs may come from annexin gene family.

Thirdly, the results of KEGG pathway showed that in most functional groups, the number of DEGs was larger in Y_Y compared to R_Y (Fig. 4), implying that *Huangguogan* has more complex regulatory networks to deal with etiolation. These pathways were mainly over-represented in amino acid metabolism (KO 00280, KO 00250, KO 00310 and KO 00330). There were 32 DEGs in Valine, leucine and isoleucine degradation pathway (KO 00280) of R_Y library, while the number was 60 in Y_Y. In addition, 15 and 22 DEGs in lysine degradation pathway (KO 00310) of R_Y and Y_Y library, respectively (Supplementary Tables S3 and S4). These findings are consistent with previous observations concerning that amino acid metabolisms are closely associated with respiration, photosynthesis, and photorespiration through interactions with carbon metabolism and regulation of chlorophyll synthesis and reducing power (NADH and NADPH)^28^,^40^,^41^. Several lines of evidence suggest that a close relationship exists between cellular redox state and amino acid metabolism^42^,^43^. GSH is at the heart of the complex antioxidant network of plants that acts to control reactive oxygen species accumulation and to facilitate appropriate cellular redox signaling and defense^44^. The increasing of the capacity for chloroplast GSH synthesis led to a general increase in amino acids such as Val, Leu, Ile, Lys, and Tyr^45^. With the treatment of glyphosate, the increase in Gln was accompanied by strongly increased pools of another major amino acid, Ala, and also by minor amino acids synthesized through shikimate-independent pathways. These included Thr, Lys, and the three branched-chain amino acids Val, Leu, and Ile^46^. Lysine acetylation, plays a major role in metabolism regulation^47^,^48^, is likely to be evolutionarily conserved^49^. A large proportion of metabolic enzymes involved in glycolysis/gluconeogenesis, citric acid cycle, and fatty acid metabolism were found to be acetylated^50^,^51^,^52^. Taken together, lysine acetylation is an essential mechanism of photosynthetic functional regulation, in a way similar to phosphorylation^53^,^54^.

Fourthly, our results showed that there were more down-regulated DEGs in photosynthesis and/or chloroplast development than up-regulated DEGs. 12 and 64 down-regulated DEGs were annotated in photosynthesis (GO: 0015979), and 4 and 10 up-regulated DEGs in photosynthesis, light reaction (GO: 0019684) in *Shiranuhi* and *Huangguogan*, respectively. The results of KEGG pathway showed that two genes (Cluster-2274.55794, Cluster-2274.57917), which were enriched in photosynthesis-antenna proteins pathway (KO 00196), were down-regulated DEGs in both R_Y and Y_Y library. In addition, the sequences of *CLA1* (Gene ID: 827230), *DET1* (Gene ID: 826609) and *GUN4* (Gene ID: 825109) of *Arabidopsis thaliana* in NCBI have been compared with transcriptome data by sequence alignment. The percentage of identical matches between *GUN4* and Cluster-2274.54336 was 67.467%, the bit score was 123, and the expect value (e-value) was 1.37e-26. When blasting in Citrus Sinensis Annotation Project (http://citrus.hzau.edu.cn/cgi-bin/orange/blast), found that the sequence of Cluster-2274.54336 was similar to Cs5g22660.1 (the total score was 1670, query cover was 67%, e-value was 0, and ident was 100%), which is the GUN4-like gen of *Citrus sinensis*. Hence the DEGs of etiolated seedlings contain *GUN4* (Cluster-2274.54336), which is thought to be one of the marker genes of chloroplast development. In both R_Y and Y_Y library, Cluster-2274.54336 was down-regulated, and the log2 FC were −2.3719 and −2.2419, respectively (Fig. 5).

In different varieties of citrus, leaf etiolation DEGs are consistent, but there is also exist species specific. Indicating that there are some differences in the performance of different citrus leaf etiolation.

## Conclusions

In this experiment, we analyzed the global transcriptome modification of two citrus cultivars—*Shiranuhi* and *Huangguogan*, that are differing in etiolation. 4225 and 2912 DEGs were identified from two cultivars, respectively, and functional analysis with these DEGs was performed. The results showed that the most prevalent functional groups in both genotypes were cellular carbohydrate metabolic process, oxidoreductase activity, and catalytic activity. We further analyzed these groups separately. Moreover, there was a unique gene encoding LEA 5 existed in R_Y and Y_Y. We also discussed the possible mechanism of etiolation, and the reason why were more and higher expression level DEGs in *Huangguogan*, and more abundant DEGs in most of common functional groups, highlighting the multiple gene control and complexity of etiolation response mechanism in *Shiranuhi* and *Huangguogan*. In summary, amino acid metabolism, especially Valine, leucine and isoleucine degradation, lysine degradation pathway, and some of the low expression genes of photosynthesis may play an important role in etiolation of *Shiranuhi* and *Huangguogan*. Although we cannot fully explain the molecular mechanism of etiolation, we have succeeded in specifying etiolation-dependent metabolic pathway and some key genes. The DEGs dataset will also provide some valuable candidate genes for functional analysis and other genetic studies in citrus seedling etiolation.

## Materials and Methods

### Plant materials

Two hybrid citrus cultivars seedlings tested in this study, *Huangguogan* and *Shiranuhi* (Supplementary Figure S1), were provided by Institute of Pomology and Olericulture, Sichuan Agricultural University. Seeds were presoaked for 4 h, and incubated in 25 °C for 3d, then sowed in pots filled with vermiculite and perlite (V:V = 1:1). Subsequently, these pots were transferred into a growth chamber set to 25 °C, 12 h light/12 h dark period and 50–60% relative humidity of air, and watered every two days after seedling germination. There were etiolated, multicoloured and green seedlings of each cultivar. 20 days after germination, more than 10 leaves were harvested from each kind of seedlings for each cultivar. These collected leaves were frozen in liquid nitrogen immediately and stored at −80 °C.

### RNA extraction and qualification

Total RNA was extracted with plant RNA Reagent (Invitrogen, Cat. No. 12322–012) following the manufacturer’s protocol. RNA purity was checked using the NanoPhotometer spectrophotometer (IMPLEN, CA, USA). RNA integrity was assessed using the RNA Nano 6000 Assay Kit of the Agilent Bioanalyzer 2100 system (Agilent Technologies, CA, USA).

### cDNA library preparation and transcriptome sequencing

A total amount of 1.5 μg RNA per sample was used as input material for the RNA sample preparations. Briefly, mRNA was purified from total RNA using poly-T oligo-attached magnetic beads. Fragmentation was carried out using divalent cations under elevated temperature in NEBNext First Strand Synthesis Reaction Buffer (5X). First strand cDNA was synthesized using random hexamer primer and M-MuLV Reverse Transcriptase (RNase H^−^). Second strand cDNA synthesis was subsequently performed using DNA Polymerase I and RNase H. Remaining overhangs were converted into blunt ends via exonuclease/polymerase activities. After adenylation of 3′ ends of DNA fragments, NEBNext Adaptor with hairpin loop structure were ligated to prepare for hybridization. In order to select cDNA fragments of preferentially 150~200 bp in length, the library fragments were purified with AMPure XP system (Beckman Coulter, Beverly, USA). Then 3 μl USER Enzyme (NEB, USA) was used with size-selected, adaptor-ligated cDNA at 37 °C for 15 min followed by 5 min at 95 °C before PCR. Then PCR was performed with Phusion High-Fidelity DNA polymerase, Universal PCR primers and Index (X) Primer. At last, PCR products were purified (AMPure XP system) and library quality was assessed on the Agilent Bioanalyzer 2100 system. The clustering of the index-coded samples was performed on a cBot Cluster Generation System using TruSeq PE Cluster Kit v3-cBot-HS (Illumia) according to the manufacturer’s instructions. After cluster generation, the library preparations were sequenced on an Illumina Hiseq 4000 and paired-end reads were generated.

### Quality control

Raw data (raw reads) of fastq format were firstly processed through in-house perl scripts. In this step, clean data (clean reads) were obtained by removing reads containing adapter, reads containing ploy-N and low quality reads from raw data. At the same time, Q20, Q30, GC-content and sequence duplication level of the clean data were calculated. All the downstream analyses were based on clean data with high quality.

### Gene functional annotation

Gene function was annotated based on the following databases: NR (NCBI non-redundant protein sequences), NT (NCBI non-redundant nucleotide sequences), Pfam (Protein family)^55^, KOG (Clusters of Orthologous Groups of proteins)^56^, Swiss-Prot (A manually annotated and reviewed protein sequence database)^57^, KO (KEGG Ortholog database)^58^, GO (Gene Ontology)^59^.

### Differential expression analysis

Gene expression levels were estimated by RSEM^60^ for each sample. Clean data were mapped back onto the assembled transcriptome. Readcount for each gene was obtained from the mapping results. Differential expression analysis of two groups was performed using the DESeq R package (1.10.1). DESeq provide statistical routines for determining differential expression in digital gene expression data using a model based on the negative binomial distribution. The resulting *P*-values were adjusted using the Benjamini and Hochberg’s approach for controlling the false discovery rate. Genes with an adjusted *P*-value < 0.05 found by DESeq were assigned as differentially expressed.

### GO and KEGG pathway enrichment analysis of differentially expressed genes(DEGs)

Gene Ontology (GO) enrichment analysis of the differentially expressed genes (DEGs) was implemented by the GOseq R packages based Wallenius non-central hyper-geometric distribution^61^, which can adjust for gene length bias in DEGs. We used KOBAS^62^ software to test the statistical enrichment of differential expression genes in KEGG pathways.

### Validation by qRT-PCR analysis

Leaves harvested from three independent seedlings of etiolated, multicoloured and green samples of both *Huangguogan* and *Shiranuhi* were used as three biological replicates. Total RNA was extracted with RNAiso Plus (TaKaRa, Dalian, China) and cDNA was synthesized by PrimeScript RT reagent Kit With gDNA Eraser (Takara, Dalian, China) according to the manufacturer’s instructions. 10 DEGs, in which 9 were up-regulated and 1 was down-regulated in both *Huangguogan* and *Shiranuhi* under etiolation, were randomly picked out for validation. Primers were designed using Primer3 (http://bioinfo.ut.ee/primer3-0.4.0/) and synthesized by Sangon Biotech. Details of selected genes and the sequence of primers were listed in Supplementary Table S1. All primers were amplified with no template control to make sure the amplicons were not primer dimers. Gene expression levels were normalized against the geometric mean of citrus reference gene, *Actin* (GenBank: XM 006480741.2) and calculated by 2^−ΔΔCT^ method.

## Additional Information

**How to cite this article:** Xiong, B. *et al*. Transcriptome Analyses of Two Citrus Cultivars (*Shiranuhi* and *Huangguogan*) in Seedling Etiolation. *Sci. Rep.* **7**, 46245; doi: 10.1038/srep46245 (2017).

**Publisher's note:** Springer Nature remains neutral with regard to jurisdictional claims in published maps and institutional affiliations.

## Supplementary Material

Supplementary Information

## Figures and Tables

**Table 1:** Overview of the sequencing results.

Sample	Raw Reads	Clean Reads	Clean Bases (G)	Q30 (%)	GC Content^a^ (%)	Mapped Ratio^b^ (%)
R_G_1	57012302	54988870	8.25	94.42	43.89	83.42
R_G_2	41815870	41233266	6.18	94.60	44.18	84.78
R_G_3	41604600	40730430	6.11	94.60	44.26	83.95
R_Y_1	44669938	42970570	6.45	94.44	43.43	82.89
R_Y_2	44683530	42874900	6.43	91.37	43.80	82.98
R_Y_3	46134030	44440236	6.67	92.85	43.48	83.03
R_M_1	48582150	46739478	7.01	94.29	44.12	83.44
R_M_2	56099636	53936088	8.09	94.19	44.18	83.19
R_M_3	50374534	48399808	7.26	94.30	44.28	83.12
Y_G_1	47891158	45978328	6.90	94.36	44.28	83.43
Y_G_2	47860120	46055110	6.91	94.47	44.18	83.91
Y_G_3	54082116	52015908	7.80	94.38	44.19	83.10
Y_Y_1	44331740	43467454	6.52	94.70	43.46	83.15
Y_Y_2	43958478	43359244	6.50	94.91	43.76	84.57
Y_Y_3	51729702	49978472	7.50	94.73	43.81	84.31
Y_M_1	47357694	45747250	6.86	94.48	44.41	85.39
Y_M_2	49140048	47483244	7.12	94.57	44.43	85.38
Y_M_3	45257470	43761800	6.56	94.75	44.43	85.45

R_G, *Shiranuhi* green seedlings. R_Y, *Shiranuhi* etiolated seedlings. R_M, *Shiranuhi* multicoloured seedlings. Y_G, *Huangguogan* green seedlings. Y_Y, *Huangguogan* etiolated seedlings. Y_M, *Huangguogan* multicoloured seedlings. ^a^The percentage of clean reads whose quality score was more than 30. ^b^The percentage of reads that are mapped to transcripts or unigenes in clean reads.

**Table 2:** List of the top ten genes with the highest FPKM in R_Y and Y_Y.

Gene ID	KEGG Pathway	R_Y fold (log_2_FC)	Y_Y fold (log_2_FC)
Cluster-2274.56949	protein. hypothetical protein CISIN_1g024065mg	—	5.92
Cluster-2274.55102	development. late embryogenesis abundant	—	2.13
Cluster-2274.48669	protein. sucrose galactosyltransferase	—	4.59
Cluster-2274.48671	protein. sucrose galactosyltransferase	—	4.62
Cluster-2274.39471	protein. Asparagine synthase	5.17	4.78
Cluster-2274.59829	protein. uncharacterized protein	8.36	8.21
Cluster-2274.79091	protein. hypothetical protein CISIN_1g004219mg	6.07	5.88
Cluster-2274.52612	protein. Myo-inositol oxygenase	4.29	3.80
Cluster-2274.29764	RNA. regulation of transcription	4.93	4.26
Cluster-2274.64715	not assigned. unknown	3.03	2.92
Cluster-2274.31940	protein. hypothetical protein CICLE_v10004369mg	7.27	—
Cluster-2274.31935	protein. hypothetical protein CICLE_v10004369mg	6.90	—
Cluster-2274.47716	not assigned. unknown	3.46	—
Cluster-2274.78324	protein. vacuolar transport	4.83	—

Data in bold symbolize genes shared by R_Y and Y_Y.
