# Supplementary material for: Transcriptome Analyses of Two Citrus Cultivars (Shiranuhi and Huangguogan) in Seedling Etiolation
Source: Sci Rep. 2017 Apr 7;7:46245. doi: 10.1038/srep46245 (PMC5384249; doi:10.1038/srep46245)
Supplement: Supplementary Information [file srep46245-s1.pdf]

Transcriptome Analyses of Two *Citrus* Cultivars (*Shiranuhi* and *Huangguogan*) in Seedling Etiolation

Bo Xiong[1 †], Shuang Ye[1 †], Xia Qiu[1], Ling Liao[1], Guochao Sun[2], Jinyu Luo[1], Lin Dai[1], Yi Rong[1] & Zhihui Wang[1,2]

[1]College of Horticulture, Sichuan Agricultural University, Chengdu 611130, China. [2]Institute of Pomology and Olericulture, Sichuan Agricultural University, Chengdu 611130, China.

Correspondence and requests for materials should be addressed to Z.-H.W. (E-mail: wangzhihui318@126.com)

[†] These authors contributed equally to this work.

Supplementary Figures

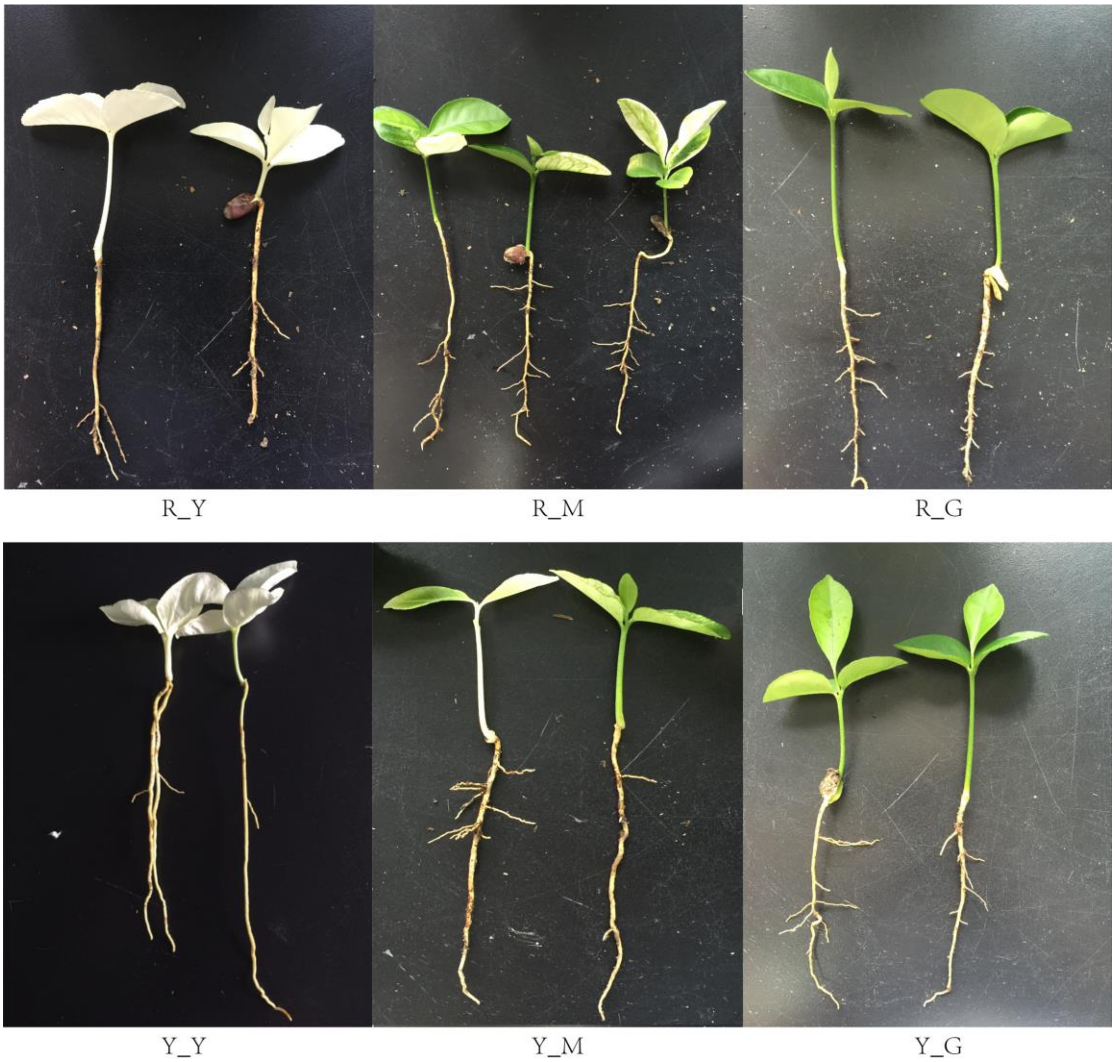

Figure S1. *Shiranuhi* (up) and *Huangguogan* (down). R_G, *Shiranuhi* green seedlings. R_Y, *Shiranuhi* etiolated seedlings. R_M, *Shiranuhi* multicoloured seedlings. Y_G, *Huangguogan* green seedlings. Y_Y, *Huangguogan* etiolated seedlings. Y_M, *Huangguogan* multicoloured seedlings.

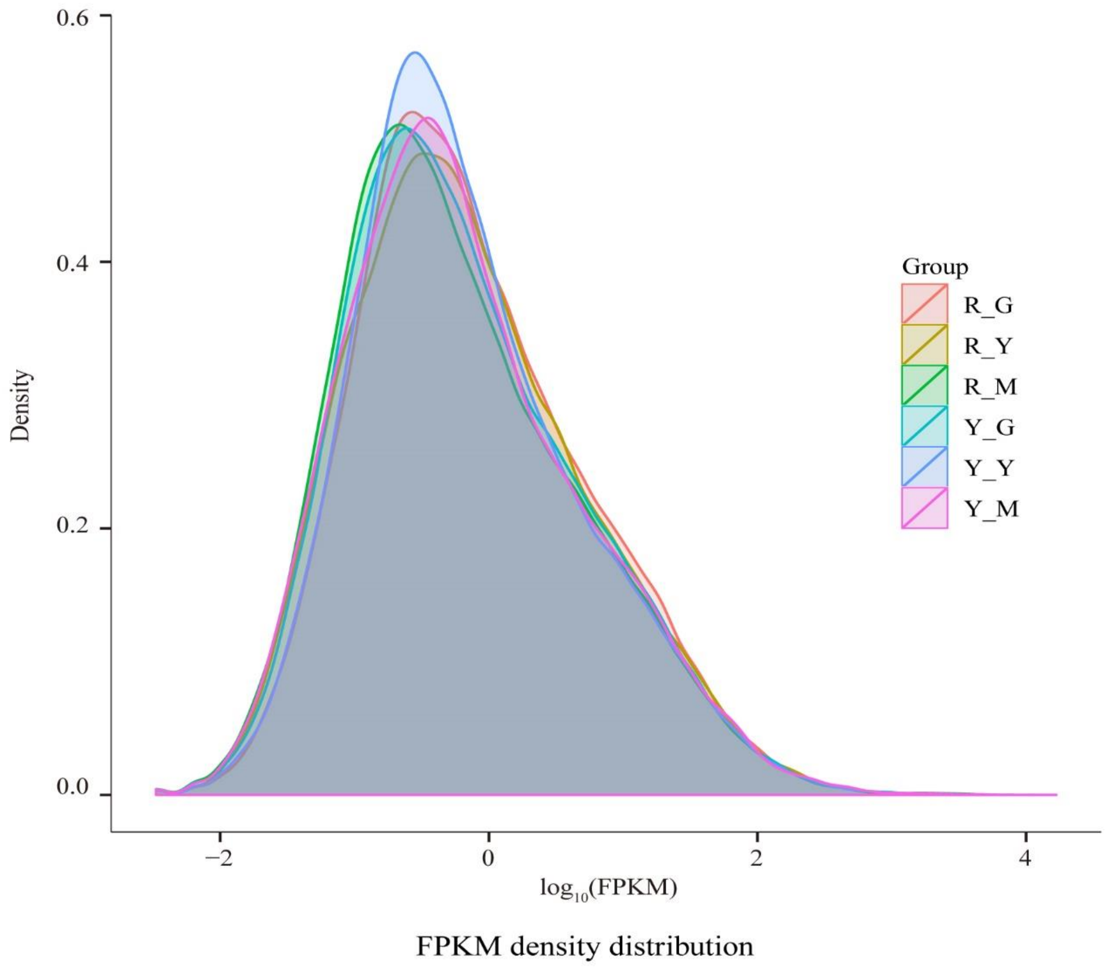

Figure S2. The FPKM density distribution of unigenes.

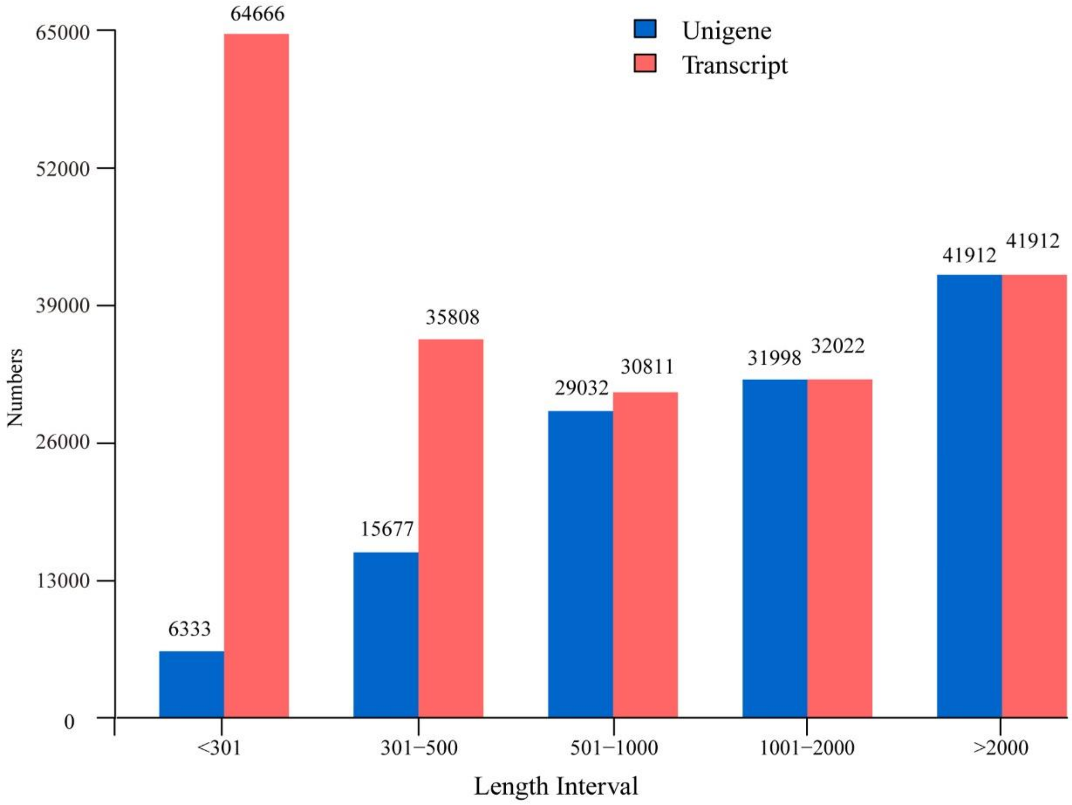

Figure S3. Unigene andTranscript length distribution.

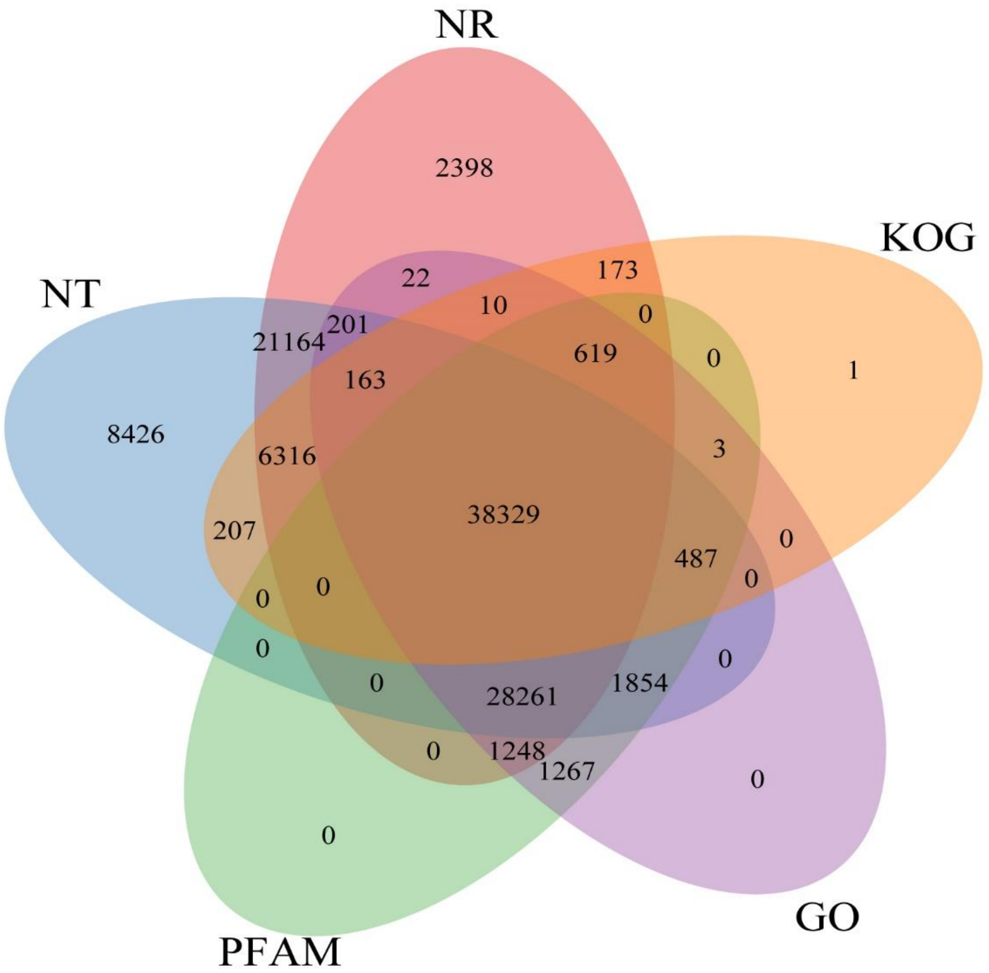

Figure S4. Annotation Venn diagram of the unigenes.

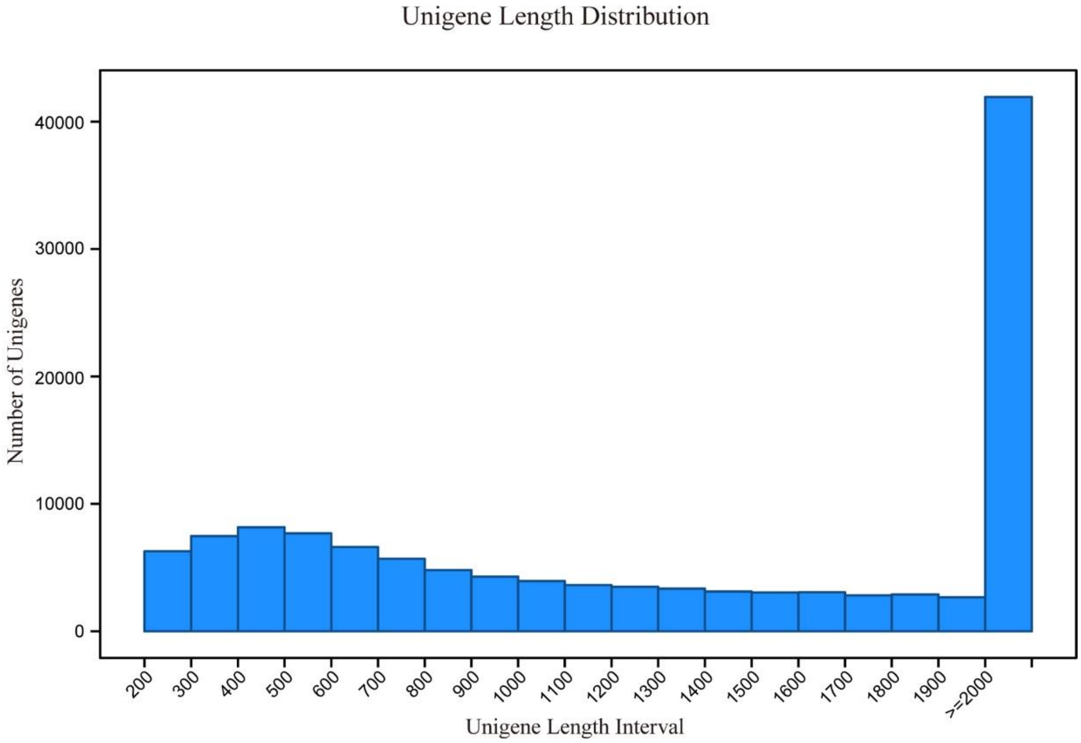

Figure S5. Overall distribution of unigenes in six libraries measured by FPKM.

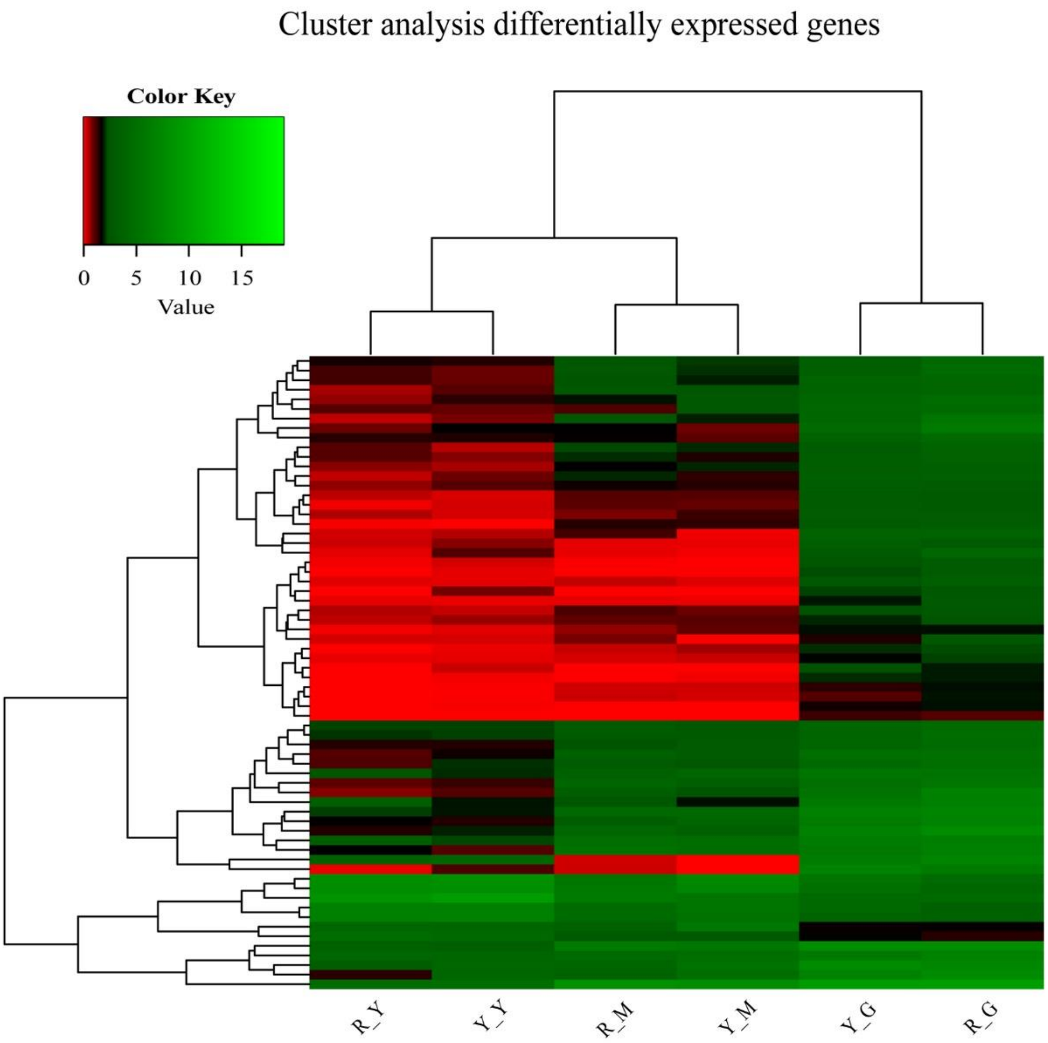

Figure S6. Hierarchical cluster analysis of DEGs in six libraries. Hierarchical cluster analysis was carried out with 66 significantly differentially expressed genes in etiolated and multicoloured seedlings of *Shiranuhi* and *Huangguogan*. Value=$\log_2$ FPKM.

Supplementary Tables

Table S1. The sequence of selected genes of primers.

Code	Gene id	Gene Length	Primer Forward	Primer Reverse
1	Cluster-2274.59829	1001	AAGCCACAAAACCACCTTTG	GAGCTACACAGCCCAGCTTC
2	Cluster-2274.31935	5764	GCTGGCCATCAAATACAGGT	CTTGGTGCATTTCCAGGTTT
3	Cluster-2274.79091	3952	ACCGGTATTGAGACCGACAG	GTCAGCATGATGAAGGCAGA
4	Cluster-2274.39471	2074	AGGAGGTGCGAGTGTAGCAT	GGTGGTGCCTTAACAGCATT
5	Cluster-2274.29764	1486	CTGGGGCTGAAAAAGTACCA	TTTTCGGTTTTACCCGACTG
6	Cluster-2274.78324	3099	ACAAAGCGTACGCGAGAGTT	ATCAAGTGGTGGAGGGACTG
7	Cluster-2274.52612	1889	GCTTCCTCAATGGGCAGTAG	AAGAGCTGCTGAAGGCAGAG
8	Cluster-2274.47716	3119	AAACGACGTCGCCGTATAAC	CAGCATCTCTCCCGACTTTC
9	Cluster-2274.64715	2370	TGGCCAATGTAGTCACCAGA	ATGGGACGACAGGTAAGTCG
10	Cluster-2274.54336	1340	ACTTGAAAGCCATCGACGAG	GGAGTTTCCTTCCCGTTCTC
Reference	*Actin* (GenBank: XM 006480741.2)	1674	CCTCACTGAAGCACCACTCA	GTGGAAGAGCATACCCCTCA

Table S2. The statistics of gene annotation success rate.

Databases	Number of Unigenes	Percentage (%)
Annotated in NR	98904	79.15
Annotated in NT	105408	84.35
Annotated in KO	42016	33.62
Annotated in SwissProt	78872	63.12
Annotated in PFAM	72068	57.67
Annotated in GO	72464	57.99
Annotated in KOG	46308	37.06
Annotated in all Databases	25194	20.16
Annotated in at least one Database	111163	88.96
Total Unigenes	124952	100.00

Table S3. The top 11 pathway of up-regulated DEGs of *Shiranuhi* enriched in KEGG

Pathway id	pathway term	1st-level(pathway)	2nd-level(pathway)	rich factor	*P*-value	$-\log_{10}$ *P*-value	gene number
KO 00280	Valine, leucine and isoleucine degradation	Metabolism	Amino acid metabolism	0.077858881	1.36E-11	10.8667	32
KO 00250	Alanine, aspartate and glutamate metabolism	Metabolism	Amino acid metabolism	0.06557377	2.42E-06	5.6169	20
KO 00310	Lysine degradation	Metabolism	Amino acid metabolism	0.066964286	5.82E-05	4.2348	15
KO 00330	Arginine and proline metabolism	Metabolism	Amino acid metabolism	0.044554455	0.000819496	3.0865	18
KO 00591	Linoleic acid metabolism	Metabolism	Amino acid metabolism	0.092105263	0.002278323	2.6424	7
KO 00340	Histidine metabolism	Metabolism	Amino acid metabolism	0.061643836	0.004047845	2.3928	9
KO 00053	Ascorbate and aldarate metabolism	Metabolism	Carbohydrate metabolism	0.070671378	1.15E-06	5.9406	20
KO 00500	Starch and sucrose metabolism	Metabolism	Carbohydrate metabolism	0.036299766	0.000139123	3.8566	31
KO 00010	Glycolysis / Gluconeogenesis	Metabolism	Carbohydrate metabolism	0.032010243	0.003658542	2.4367	25
KO 00620	Pyruvate metabolism	Metabolism	Carbohydrate metabolism	0.034305317	0.004950828	2.3053	20
KO 00910	Nitrogen metabolism	Metabolism	Energy metabolism	0.058823529	0.002073841	2.6832	11

Table S4. The top 11 pathway of up-regulated DEGs of *Huangguogan* enriched in KEGG

Pathway id	pathway term	1st-level(pathway)	2nd-level(pathway)	rich factor	*P*-value	-$\log_{10}$ *P*-value	gene number
KO 00280	Valine, leucine and isoleucine degradation	Metabolism	Amino acid metabolism	0.145985401	1.71E-22	21.7661	60
KO 00250	Alanine, aspartate and glutamate metabolism	Metabolism	Amino acid metabolism	0.091803279	1.53E-06	5.8142	28
KO 00310	Lysine degradation	Metabolism	Amino acid metabolism	0.098214286	9.71E-06	5.0128	22
KO 00290	Valine, leucine and isoleucine biosynthesis	Metabolism	Amino acid metabolism	0.132653061	8.34E-05	4.0791	13
KO 00340	Histidine metabolism	Metabolism	Amino acid metabolism	0.102739726	0.000214237	3.6691	15
KO 00330	Arginine and proline metabolism	Metabolism	Amino acid metabolism	0.066831683	0.000237851	3.6237	27
KO 00053	Ascorbate and aldarate metabolism	Metabolism	Carbohydrate metabolism	0.098939929	6.02E-07	6.2205	28
KO 00500	Starch and sucrose metabolism	Metabolism	Carbohydrate metabolism	0.053864169	0.000123257	3.9092	46
KO 00040	Pentose and glucuronate interconversions	Metabolism	Carbohydrate metabolism	0.074204947	0.000425509	3.3711	21
KO 00903	Limonene and pinene degradation	Metabolism	Metabolism of terpenoids and polyketides	0.146551724	1.53E-06	5.8142	17
KO 00071	Fatty acid degradation	Metabolism	Lipid metabolism	0.077348066	2.16E-05	4.6647	28
